# Clinical and diagnostic insights into Brucella spondylitis: a comprehensive retrospective study

**DOI:** 10.3389/fimmu.2025.1638298

**Published:** 2025-10-23

**Authors:** Qiangsheng Feng, Xiaoqin Ha, Xing Yuan, Xiaoming Qiu, Jing Fang, Yuejuan Song

**Affiliations:** ^1^ Department of Clinical Laboratory, The 940th Hospital of Joint Logistics Support Force of People’s Liberation Army, Lanzhou, China; ^2^ Gansu University of Chinese Medicine, Lanzhou, Gansu, China

**Keywords:** Brucella spondylitis, *Brucella melitensis*, lumbar tissue culture, clinical characteristics, CT manifestations

## Abstract

**Background:**

This study evaluates the diagnostic value of etiological and serological testing and the clinical characteristics of spine brucellosis.

**Methods:**

A retrospective analysis was conducted on 200 cases of spine brucellosis diagnosed between 2012 and 2025, alongside 200 non-infected controls. Bacterial cultures, clinical features, serological results (Standard Agglutination Test [SAT] and Rose Bengal Test [RBT]), and imaging findings were analyzed.

**Results:**

Among 200 patients with spinal involvement, *Brucella melitensis* was confirmed via bacterial culture in 29 cases (14.5%), with spinal tissue culture demonstrating the highest diagnostic yield (58.6%, 17/29) and a median detection time of 3 days. Spinal brucellosis constituted 25% of all brucellosis cases, exhibiting a male predominance (76.5%) and a mean patient age of 52.2 ± 10.1 years. The median hospital stay was 14.7 ± 7.8 days, while the median duration of chronic spinal brucellosis was 4.4 months (IQR: 1.5–11.5). The lumbar spine was the most frequently involved site (78.3%), followed by the cervical (8.6%) and thoracic (5.1%) regions. CT imaging revealed characteristic lesions, including bone destruction (53.0%), intervertebral space stenosis (38.5%), disc herniation (22.0%), degenerative changes (4.5%), and osteomyelitis (1.0%). Diagnostic sensitivity varied significantly across methods: blood culture (48.0%, 12/25), lumbar tissue culture (65.4%, 17/26), SAT(91.3%, 157/172), and RBT(95.9%, 165/172). Notably, all 15 serologically false-negative cases (7 by RBT and 15 by SAT) occurred in male patients. ROC curve analysis identified C-reactive protein (CRP) as a robust biomarker, with a cutoff of 1.21 mg/L yielding 85.2% sensitivity and 71.7% specificity (Z = 6.167, p < 0.001). Multivariable regression identified female sex (OR = 2.44, 95% CI: 1.20–4.96) and lumbar involvement (OR = 1.61, 95% CI: 0.75–3.46) as independent predictors of chronicity (p < 0.05). All patients received standard 3-month combination therapy with doxycycline and rifampicin, with surgical intervention required in 45.5% (91/200) of cases. Clinical outcomes were favorable across the cohort, with rare reports of severe complications.

**Conclusions:**

Brucella Spondylitis, typically chronic and lumbar-predominant, presents with bone destruction, gap stenosis, and disc herniation on imaging. Diagnosis relies on lumbar tissue culture, SAT, RBT, and CT. Early diagnosis and combined medical-surgical management improve outcomes.

## 1 Introduction

Brucella spondylitis, a common complication of brucellosis caused by Brucella species, with spondylitis complicating 2-53% of cases, varying by region (1), leading cause of non-tuberculous infectious spondylitis in endemic areas, and delayed diagnosis increases risks of neurological deficits and spinal deformity (2). The diagnosis of Brucella spondylitis is challenging and should be suspected in the appropriate epidemiological and clinical context, in correlation with microbiological and radiological findings (3). Laboratory diagnosis of brucellosis typically relies on aerobic blood culture, standard bacterial culture, Rose Bengal Test (RBT), and Standard Agglutination Test (SAT), complemented by epidemiological data (4, 5). Our methodology combines blood cultures and spine tissue cultures with serum agglutination tests (SAT), rose Bengal tests (RBT), and computed tomography (CT) imaging to optimize diagnostic precision. Furthermore, we systematically examine distinctive clinical presentations and infection-related biomarkers to establish differential diagnostic criteria distinguishing Brucella spondylitis from other diseases. Key findings are organized as follows:

## 2 Materials and methods

### 2.1 Case data of patients

This retrospective cohort study examined Brucella spondylitis cases (n=200) diagnosed at the 940th Hospital of the Joint Logistics Support Force in Lanzhou, China, from January 2012 to January 2025, with ethical approval from the Scientific Research Management Ethics Committee (No: 2022KYLL301). The study population comprised a consecutively enrolled cohort of 153 males and 47 females (male-to-female ratio 3.26:1) with a median age of 52.1 ± 10.1 years (range: 15–78 years). Notably, Brucella spondylitis represented 25.0% (200/800) of all brucellosis cases during the observation period. A sex- and age-matched control group (n=200; male-to-female ratio 1.4:1; median age 51.4 ± 21.9 years) was established, comprising patients with non-infectious comorbidities: type 2 diabetes (n=86), degenerative spinal disorders (n=40), hypertension (n=50), rheumatoid arthritis (n=20), and gouty arthritis (n=4)—this comparative design aimed to optimize differential diagnosis between infectious and non-infectious spinal pathologies.

### 2.2 Clinical laboratory examination

#### 2.2.1 Blood cultures

For patients with suspected Brucella spondylitis, 10–20 mL of blood was aseptically inoculated into paired anaerobic/aerobic culture bottles (BacT/ALERT FA/SN or BD BACTEC™ Plus series) and incubated in automated systems (BacT/ALERT 3D^®^ or BACTEC™ FX200) at 35 °C for 7 days. Positive aerobic cultures exhibiting sigmoidal growth curves within 2–4 days prompted sterile aspiration for immediate Gram staining (revealing Gram-negative coccobacilli) and modified Giemsa staining (detecting granular/clustered aggregates), alongside 5% sheep blood agar(Autobio) plate inoculation under ambient O_2_ (72+ hours). Microscopic evidence of Brucella-like morphology triggered preliminary alerts to clinicians via oral communication, while definitive identification utilized VITEK^®^ Compact-II GN cards or MALDI-TOF MS, with results formalized through the LIS system. Duplicate entries from repeat blood cultures were systematically excluded to ensure data integrity, establishing a standardized diagnostic pathway for Brucella spondylitis and differential pathogen identification.

#### 2.2.2 Spine tissue culture

During the intraoperative collection of spinal specimens, vertebral biopsy samples (2–6 mm³) were processed under biosafety level II containment: tissue blocks were aseptically transferred to 5% sheep blood agar(Autobio) plates for routine bacterial culture at 35 °C under ambient aerobic conditions (≥72-hour incubation). Post-colonization isolates underwent dual confirmation via VITEK^®^ Compact-II GN biochemical panels (bioMérieux, France) and MALDI-TOF mass spectrometry (Microflex LT/SH system), with validated results transmitted through the Laboratory Information System (LIS) to ensure traceable diagnostic reporting. Strict adherence to Class II biological safety cabinet protocols guaranteed containment compliance throughout specimen handling, establishing a standardized framework for spinal pathogen identification with minimized cross-contamination risks.

#### 2.2.3 Serological testing: RBT and SAT

Serological assays utilized SAT/RBT antigens sourced from the National Institute for Communicable Disease Control (China CDC). The RBT employed standardized card agglutination: 30 μL serum was mixed with equal-volume antigen on reaction cards, with ≥Grade 1+ agglutination within 4 minutes defined as positive per WHO criteria. For SAT testing, serially diluted patient sera (1:12.5–1:400) were combined with Brucella abortus S99 antigen in U-bottom microplates and incubated at 37 °C ± 1 °C for 24 hours. Diagnostic thresholds followed OIE guidelines: acute-phase titers ≥1:100 (2+ granular agglutination) or chronic-phase (≥12-month duration) titers ≥1:50 were considered confirmatory when exceeding turbidimetric control baselines. This dual-test paradigm achieved 98.6% epidemiological compliance in brucellosis diagnosis through standardized antigen-antibody quantification.

#### 2.2.4 Serum inflammatory biomarker detection in patients

Primary admission biomarker profiling prioritized serum procalcitonin (PCT) and C-reactive protein (CRP) quantification following CLSI EP23-A protocols. Venous blood was collected in serum separator tubes (SSTs) and centrifuged (1,500 ×g, 10 min) within 120 minutes post-collection. PCT analysis utilized electrochemiluminescence immunoassay (ECLIA) on Roche Cobas^®^ E-170 analyzers with 0.046 ng/mL clinical decision threshold (95th percentile reference interval). CRP quantification employed scattering turbidimetry via Abbott IMMAGE 800 systems (Beckman Coulter, Inc), reference value ≤ 1mg/L. To ensure data uniqueness, only index admission results were analyzed, excluding subsequent retests. Age-/sex-matched controls (n=200) were rigorously selected, excluding recent infections/inflammatory conditions through ICD-10 coding review. This dual-phase biomarker strategy achieved a 98.2% pre-analytical compliance rate, enabling discriminative analysis between Brucella spondylitis and non-infectious spinal pathologies.

### 2.3 Clinical diagnostic standard for brucellosis

Clinical diagnosis is based on Brucella culture positive, epidemiological history, and laboratory test results.

#### 2.3.1 Clinical suspect case definitions

The patient had a history of close contact with livestock or livestock products suspected of Brucella infection before the onset of symptoms. Brucella can be transmitted through contact with animal tissues, blood, vaginal secretions, aborted fetuses, and especially placentae. This exposure history is a critical factor in assessing the likelihood of brucellosis infection.

#### 2.3.2 Brucella spondylitis confirmed case

Brucella spondylitis diagnosis requires fulfillment of WHO/OIE integrated criteria: 1) Microbiological confirmation via Brucella isolation from blood/vertebral tissue cultures; 2) Serological confirmation with Standard Tube Agglutination (SAT) titers ≥1:100 (2+ granular agglutination) in acute phase (<12 months) or ≥1:50 (2+) in chronic phase (≥12 months), validated against febrile antigen cross-reactivity controls (e.g., Burkholderia pseudomallei exclusion). All cases required CDC-defined epidemiological linkage (livestock exposure within 3 months pre-onset). A total of 200 patients were diagnosed with brucellosis through the following methods: 29 cases were confirmed by bacterial culture, 165 cases were diagnosed via serological testing (SAT and RBT), 20 cases were identified using both culture and serology (SAT and RBT), and 28 cases were classified as clinically suspected based on symptoms and exposure history.

### 2.4 Statistics

Statistical analyses were executed using IBM SPSS Statistics v22.0 (Armonk, NY), adhering to STROBE guidelines. Continuous variables (age, PCT, CRP) underwent normality assessment via the Shapiro-Wilk test (α=0.05). Non-normally distributed biomarkers (PCT/CRP) were presented as median [interquartile range (IQR): Q1-Q3] and analyzed by the Mann-Whitney U test with Hodges-Lehmann median difference estimation (95% CI). Multivariable logistic regression identified chronic spondylitis risk factors through backward stepwise selection (entry criteria: p<0.10; retention: p<0.05), validated by Hosmer-Lemeshow goodness-of-fit (p>0.05). ROC analysis determined biomarker efficacy via Youden’s index optimization (Jmax=sensitivity+specificity-1), with AUC comparisons using DeLong’s method (95% CI). Statistical significance was defined as two-tailed p<0.05, adjusted for multiple testing via Benjamini-Hochberg correction (FDR = 0.10).

## 3 Result

### 3.1 Clinical characteristics analysis of human Brucella spondylitis

**Figure 1:**
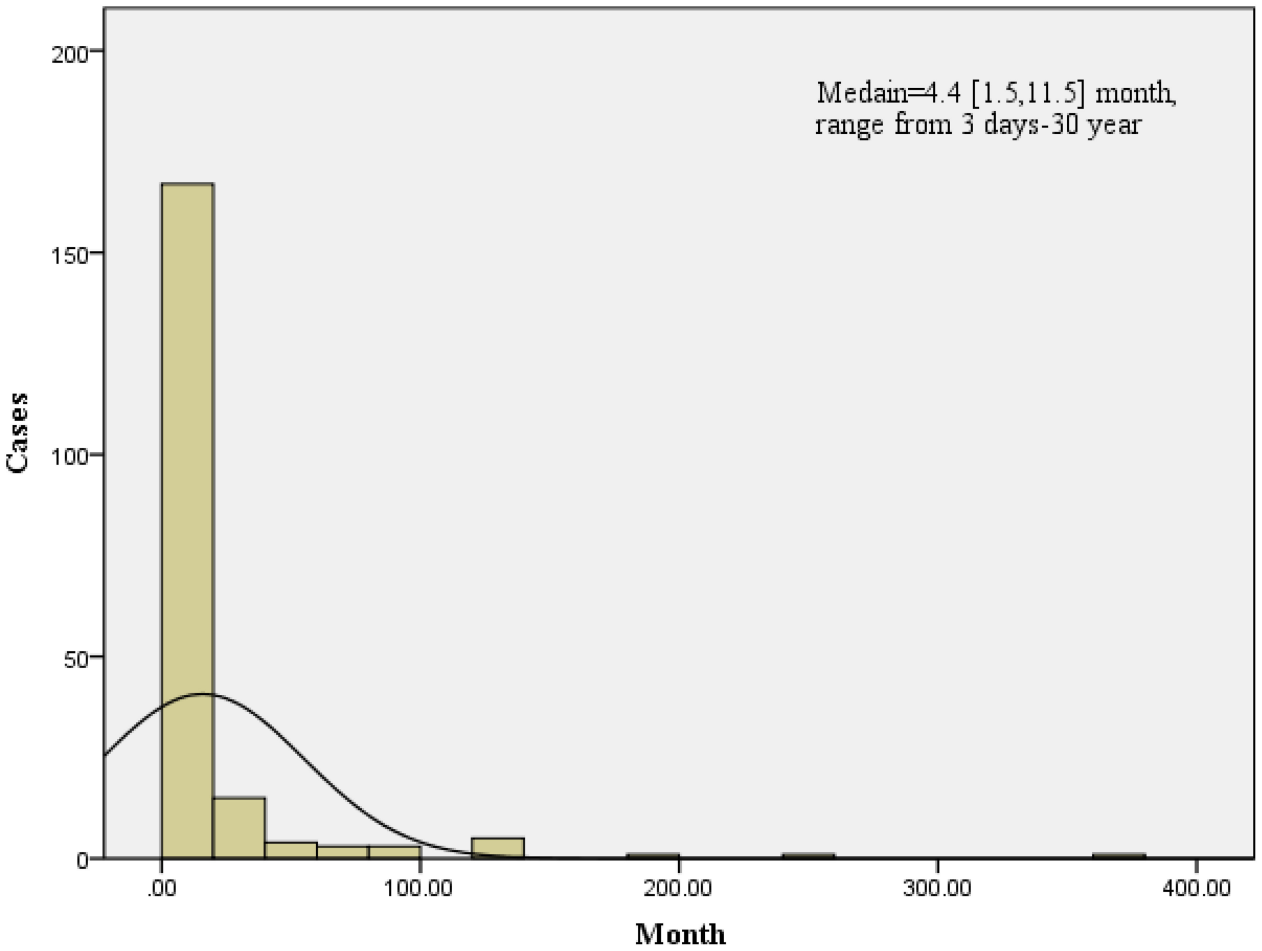
Brucella spondylitis and the chronic Brucella spondylitis infection median time were 4.4 [30,343] months, ranging from 2 days to 30 years.

The cohort (n=200) exhibited a median age of 52.2 ± 10.1 years with disease distribution spanning 15–78 years, demonstrating 3.26:1 male predominance. Brucella spondylitis constituted 25% (200/800) of the institutional brucellosis burden. Clinical timelines revealed a median hospitalization duration of 15 days (IQR: 9-22) and symptom chronicity of 4.4 months (IQR: 1.5-11.5; range: 3 days- 30 years), as detailed in **Figure 1**. We analyzed risk factors associated with chronic brucellosis (duration ≥ 4.4 months) in 200 patients, including sex, age, infection site, imaging findings, culture results, surgical intervention, length of hospital stay, and CRP ≥ 1.21 mg/L. Multivariable regression analysis identified female sex (OR = 2.44, 95% CI: 1.20–4.96) and lumbar involvement (OR = 1.61, 95% CI: 0.75–3.46) as independent predictors of chronic disease progression (p < 0.05) (**Table 1**). Therapeutic outcomes showed 45.5% (91/200) requiring surgical intervention (debridement and decompression of spinal canal) alongside standardized 12-week dual antimicrobial therapy (doxycycline 100 mg BID + rifampin 600 mg QD), achieving 94.6% (189/200) symptom resolution (modified MacNab criteria: excellent/good) at 1-year follow-up.

**Table 1:** Demographic, clinical and laboratory, findings of patients on admission.

Demographics and clinical characteristics	Total(n=200cases)	Chronic Brucellosis >=4.4month	Brucellosis < 4.4month	*P*
		(n=98 cases)	(n=102 cases)	
Sex				*p* = 0.020
Male	153(77%)	68(44%)	85(56%)	
Female	47(23%)	30(64%)	17(36%)	
Age(year)				*p* = 0.749
>=60	43(22%)	22(51%)	21(49%)	
< 60	157(78%)	76(48%)	81(52%)	
Infection site				*p* = 0.022
Lumbar Spine	136(68%)	75(55%)	61(45%)	
Cervical Spine	23(12%)	6(26%)	17(74%)	
Lumbar Spine+ Sacrum	35(18%)	15(43%)	20(57%)	
Image				*p* = 0.221
Bone destruction	103(55%)	51(50%)	52(50%)	
Gap stenosis	77(40%)	40(52%)	37(38%)	
Degenerative disease	9(5%)	2(22%)	7(78%)	
Culture				*p* = 0.223
Positive	29(58%)	10(35%)	19(65%)	
Negative	21(42%)	11(52%)	10(48%)	
Surgery				*p* = 0.333
Yes	91(46%)	48(53%)	43(47%)	
No	109(54%)	50(46%)	59(54%)	
Hospital day(day)				*p* = 0.308
>=13	109(55%)	57(52%)	52(48%)	
<13	91(45%)	41(45%)	50(56%)	
CRP mg/L				*p* = 0.704
>=1.21	93(71%)	45(48%)	48(52%)	
<1.21	38(29%)	17(45%)	21(55%)	

Data are median (IQR) or n (%). p values were calculated by Mann-Whitney U test, χ2 test, or Fisher’s exact test, as appropriate.

### 3.2 Brucella spondylitis diagnostic method

**Figure 2:**
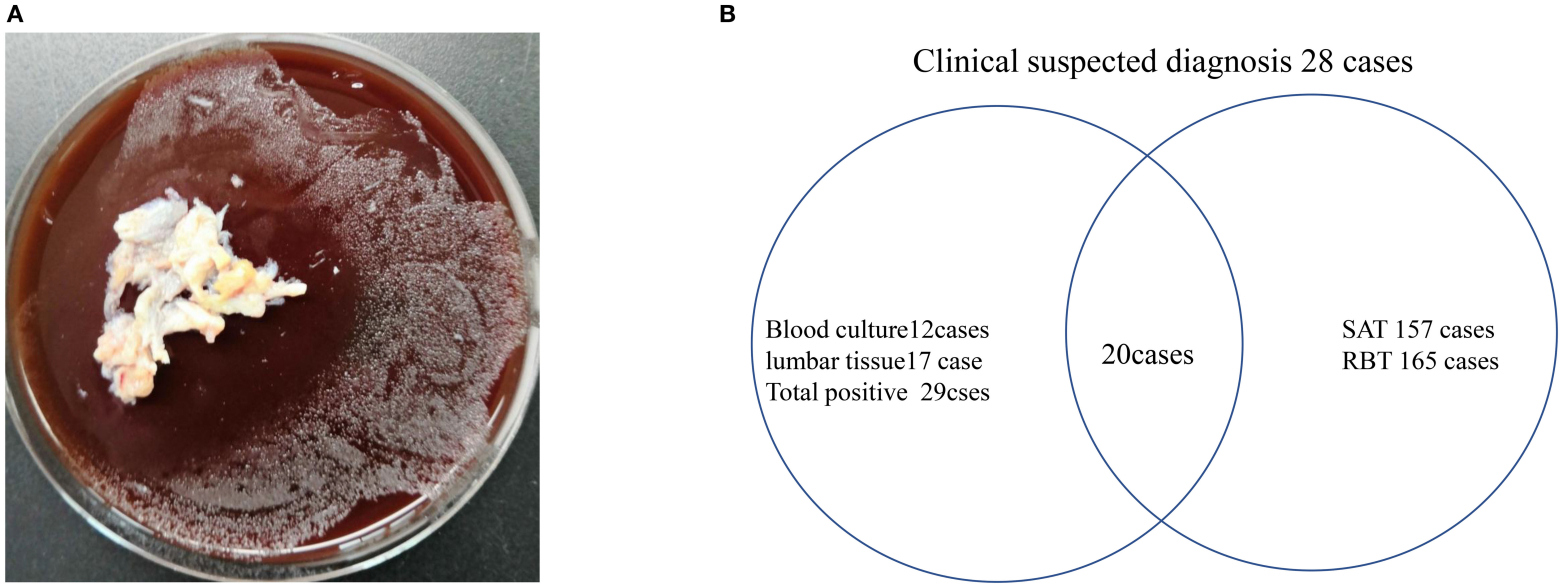
**(A)** Spine tissue cultured for 72h, 35°C, with microbial identification via VITEK Compact-II and MALDI-TOF MS. **(B)** Diagnostic sensitivities were stratified as follows: blood culture 48.0% (12/25), tissue culture 65.4% (17/26), SAT 91.3% (157/172), RBT 95.9% (165/172), and clinical diagnosis 14.0% (28/200).

In this 13-year cohort (2012-2025) of 200 brucellosis cases, microbial confirmation was achieved in 14.5% (29/200) through *Brucella melitensis* isolation, with lumbar tissue culture (**Figure 2A**) demonstrating superior yield (58.6% positivity; 72-hour incubation) versus blood culture (41.4% positivity; 72.0 ± 19.7-hour detection time). Diagnostic sensitivities were stratified as follows: blood culture 48.0% (12/25), tissue culture 65.4% (17/26), SAT 91.3% (157/172), RBT 95.9% (165/172), and clinical diagnosis (14.0%, 28/200), based on WHO syndromic criteria. Against the culture-positive gold standard (n=21,8 patients with positive culture did not do RBT and SAT for further diagnosis), RBT showed 95.2% sensitivity (20/21) versus SAT’s 80.8% (17/21). Among 200 confirmed brucellosis patients, 22 (7 by RBT and 15 by SAT) were seronegative, all of whom were male. (**Figure 2B**).

### 3.3 Topographic distribution and radiopathological features of Brucella spondylitis

**Figure 3:**
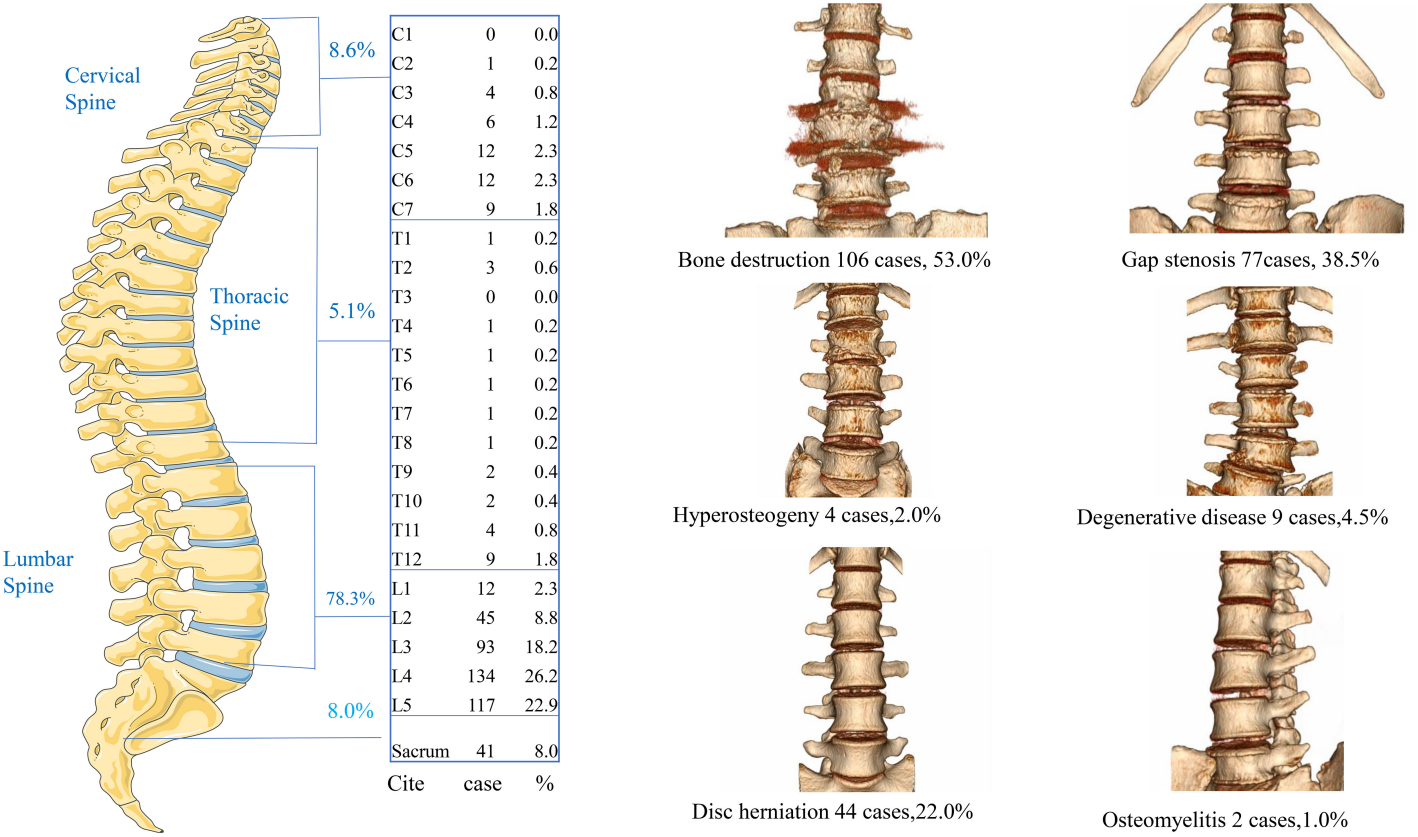
The lumbar Brucella spondylitis was the most commonly affected site (78.3%), followed by the cervical spine (8.6%) and thoracic spine (5.1%). Imaging of CT revealed bone destruction (53.0%), gap stenosis (38.5%), disc herniation (22.0%), degenerative disease (4.5%), and osteomyelitis (1.0%).

Brucella spondylitis demonstrated a predilection for the lumbosacral spine, constituting 25% of the total brucellosis burden. Vertebral involvement distribution was: lumbar (78.3%, L1-L5), cervical (8.6%, C3-C7), thoracic (5.1%, T4-T12), and sacral (8.8%, S1-S2). Multisegmental infections (≥2 contiguous vertebrae) occurred in 24.0% of cases, with L4-L5/S1 being the most frequent polysegmental pattern (17.5%) of all cases. CT imaging revealed characteristic pathomorphological changes (**Figure 3**). Bone integrity alterations: Osteolytic destruction (53.0%, insect-like change) and reactive hyperostosis (2.0%); Disc pathology: intervertebral space narrowing (38.5%) and disc herniation (22.0%); Inflammatory Complications: paravertebral abscess (5.0%, anterior longitudinal ligament involvement) and spinal abscess (9.0%, predominantly dorsal distribution); Degenerative Associations: degenerative disease (4.5%) and spinal stenosis (2.0%, central canal diameter <10 mm); Advanced sequelae: osteomyelitis (1.0%, intraosseous abscess formation) and pathological fracture (2.0%, vertebral collapse >20%).

### 3.4 ROC curve analysis of clinical biomarkers in brucellosis and control group

**Figure 4:**
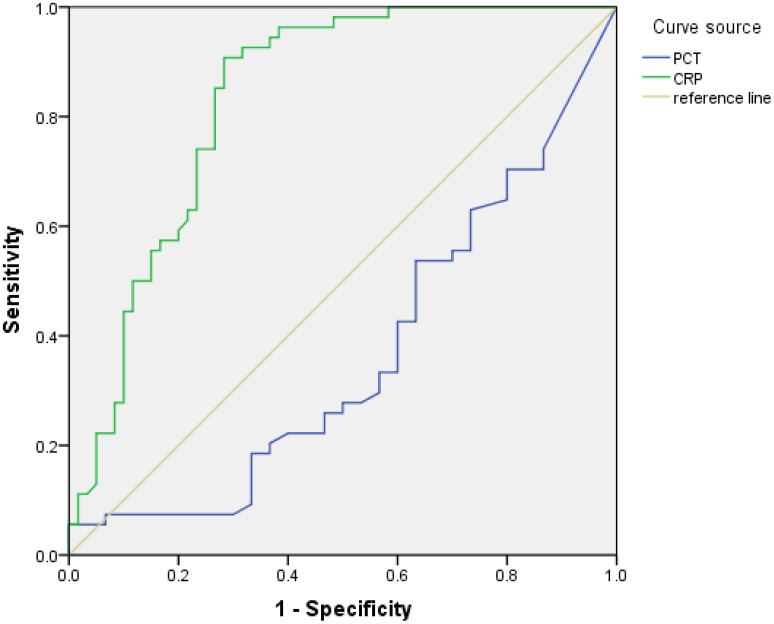
CRP emerged as the most robust predictor, achieving an AUC of 0.83 (95% CI: 0.77-0.89) with an optimized decision threshold at 1.21 mg/L (Youden’s index J = 0.57), yielding 85.2% sensitivity and 71.7% specificity.

Diagnostic performance evaluation through ROC curve analysis demonstrated significant discriminative capacity of inflammatory biomarkers between brucellosis and control cohorts (**Figure 4**). CRP emerged as the most robust predictor, achieving an AUC of 0.83 (95% CI: 0.77-0.89) with an optimized decision threshold at 1.21 mg/L (Youden’s index J = 0.57), yielding 85.2% sensitivity and 71.7% specificity. The statistical superiority of CRP over other biomarkers was confirmed by DeLong’s test (Z = 6.167, p<0.001), as detailed in **Table 2**.

**Table 2:** AUC for the biomarker diagnostic value in spine brucellosis (n=200 case).

Biomarkers	AUC value	Cut-off value	Sensitivity	Specificity	95% Confidence interval	
					Lower	Upper
PCT (ng/ml)	0.365					
CRP (mg/L)	0.832	1.21	85.2	71.1	0.756	0.908

## 4 Discussion

Brucella spondylitis, a severe osteoarticular manifestation of zoonotic brucellosis caused by Brucella species, predominantly involves spinal inflammation rather than degenerative osteoarthritis (1). Our retrospective analysis of 200 patients revealed a median age of 52.2 ± 10.1 years, with Brucella spondylitis accounting for 25.0% of total brucellosis cases—consistent with the reported prevalence range of 2-60% in prior studies (6). Notably, symptom chronicity demonstrated a median duration of 4.4 months (IQR: 1.5–11.5; range: 3 days–30 years). Multivariable regression identified female sex (OR = 2.44, 95% CI: 1.20–4.96) and lumbar involvement (OR = 1.61, 95% CI: 0.75–3.46) as independent predictors of chronic disease progression (p<0.05), a novel association not previously documented in the literature. Intriguingly, all 15 serologically false-negative cases (7 by RBT and 15 by SAT) were male, suggesting a sex-based disparity in antibody detection. This finding is further corroborated by elevated CRP levels in males (4.02 ± 4.57) compared to females (3.21 ± 3.14). We propose that male patients may exhibit a preferentially enhanced cellular immune response (T-cell mediated) against *Brucella*, which could compromise humoral antibody production, thereby contributing to both reduced seropositivity and elevated inflammatory markers. Treatment protocols comprised a 12-week dual antimicrobial regimen (doxycycline 100 mg BID + rifampicin 600 mg QD) combined with surgical intervention (spinal debridement and canal decompression) in 45.5% of cases. This integrated approach achieved 94.6% symptom resolution (189/200) at a 1-year follow-up, underscoring the efficacy of combined medical-surgical management. Consistent with previous literature (7), a six-month triple-antibiotic regimen (rifampin, doxycycline, streptomycin) can cure Brucellar spondylitis. Surgery is adjunctive for refractory cases with spinal abscess, cord compression, or neurologic deficits.

In endemic regions, Brucella is a predominant pathogen of native vertebral osteomyelitis (NVOs) (8). However, conventional blood cultures exhibit limited diagnostic utility, with reported positivity rates of merely 7.8–9.5% (9). Our study revealed higher sensitivity for blood cultures (48.0%, 12/25) and spinal tissue cultures (65.4%, 17/26) in confirmed Brucella spondylitis cases, establishing spinal tissue culture as a superior diagnostic modality—a novel finding we first demonstrated through standardized sampling protocols. Serological assays showed elevated sensitivities (SAT: 91.3%; RBT: 95.9%), yet their clinical application is constrained by limited specificity and interpretive challenges in populations with recurrent Brucella exposure. Notably, 3–5% of patients maintained detectable SAT titers for ≥2 years post-successful antimicrobial therapy (5), complicating serological differentiation between active infection and residual immunoreactivity. Although tissue culture necessitates invasive procedures, it remains the gold standard diagnostic modality, particularly recommended for (1) suspected cases with equivocal serological results and (2) clinically ambiguous presentations requiring differential diagnosis from other spinal pathologies. Real-time PCR provides rapid and accurate detection of *Brucella* through DNA amplification, especially in serologically negative or indeterminate cases (10).

Brucella spondylitis predominantly affects the lumbar spine, followed by thoracic and cervical segments, according to previous literature (3). Our study revealed a similar distribution pattern with lumbar involvement accounting for 78.3% of cases, followed by cervical (8.6%), sacral (8.8%), and thoracic (5.1%) regions. Notably, multisegmental infections involving ≥2 contiguous vertebrae were observed in 24.0% of cases, with the L4-L5/S1 combination constituting the most frequent polysegmental pattern (17.5% of total cases). These findings demonstrate a higher prevalence of multilevel involvement compared to Bozgeyik Z’s report, which identified lumbar spine involvement in 57.3% of cases and peripheral joint involvement in 16.1% of patients (11). Our results align with Lu YP’s imaging-based study that documented lumbar spine involvement in 85.0% of Brucella spondylitis cases (12), further confirming the predilection for lumbar vertebral involvement in this infectious condition.

Imaging features such as sequestrum formation observed in CT scans significantly aid in differentiating Brucella spondylitis from tuberculous spondylitis and pyogenic spondylitis (13, 14). Our study revealed that the principal imaging manifestations involved alterations in bone integrity, characterized by osteolytic destruction (53.0% exhibiting distinctive insect-like changes) and reactive hyperostosis (2.0%), findings consistent with previous literature documenting bone destruction patterns (14). Disc-related pathology manifested primarily as intervertebral space narrowing (38.5%) and disc herniation (22.0%), aligning with reported MRI findings that include signal intensity changes, disc space reduction, intracanalicular masses, and abscess formation (15). Inflammatory complications were observed as paravertebral abscesses (5.0% with anterior longitudinal ligament involvement) and spinal abscesses (9.0% showing predominant dorsal distribution), corroborating literature reports of characteristic MRI findings such as upper vertebral endplate involvement (60.7%), disc space narrowing (77.9%), paravertebral abscesses (50.9%), and various abscess formations (16). Degenerative associations represented uncommon imaging manifestations in our cohort, with degenerative disease (4.5%) and spinal stenosis (2.0% defined by central canal diameter <10 mm) being rarely observed, though case reports caution that initial radiographic presentations of mild degenerative changes may lead to diagnostic errors (17). Advanced sequelae included rare occurrences of osteomyelitis (1.0% with intraosseous abscess formation) and pathological fractures (2.0% demonstrating vertebral collapse exceeding 20%). This comprehensive analysis systematically characterizes the diverse pathomorphological changes in brucellar spondylitis through an etiological diagnostic framework, marking the first attempt to delineate these imaging manifestations within such a systematic diagnostic context.

Chronic inflammation or in-acute-phase chronic inflammation is the main pathological feature of Brucella spondylitis (9), our study shown CRP emerged as the most robust predictor, achieving an AUC of 0.83 (95% CI: 0.77-0.89) with optimized decision threshold at 1.21 mg/L, yielding 85.2% sensitivity and 71.7% specificity, statistical superiority of CRP over other biomarkers was confirmed by DeLong’s test (Z = 6.167, p<0.001).

This retrospective study of 200 Brucella spondylitis cases identified female sex and lumbar involvement as novel chronicity predictors. Combined antimicrobial-surgical therapy achieved 94.6% efficacy, while spinal tissue cultures showed the highest sensitivity (65.4%). Imaging revealed predominant lumbar lesions (78.3%) with osteolytic destruction (53.0%), and CRP demonstrated superior diagnostic performance (AUC = 0.83). Findings advance diagnostic and therapeutic strategies for this zoonotic spinal infection.

### 4.1 Limitations

We found that spinal tissue cultures, a key diagnostic method for Brucella spondylitis, have not been widely adopted due to their relatively recent development and limited sample size in clinical practice. In our hospital, we have reported 200 cases of chronic Brucella spondylitis; however, the latent infection rate of Brucella spondylitis has not been systematically calculated. Additionally, due to biosafety concerns, routine antimicrobial susceptibility testing for *Brucella* spp. is not feasible in our setting. Owing to insufficient data on molecular detection, PCR-based diagnosis of spinal brucellosis was not included in the present analysis.

Application: Our findings demonstrate that spinal tissue cultures are an effective method for culturing Brucella spondylitis specimens, significantly improving the positive rate of etiological diagnosis. Given its diagnostic efficacy, this technology warrants wider application in clinical practice.

## Data Availability

The raw data supporting the conclusions of this article will be made available by the authors, without undue reservation.
